# *Icex*: Advances in the automatic extraction and volume calculation of cranial cavities

**DOI:** 10.1111/joa.13843

**Published:** 2023-02-11

**Authors:** Costantino Buzi, Antonio Profico, Ce Liang, Roman H. Khonsari, Paul O'Higgins, Mehran Moazen, Katerina Harvati

**Affiliations:** ^1^ DFG Centre of Advanced Studies ‘Words, Bones, Genes, Tools’ Eberhard Karls University of Tübingen Tübingen Germany; ^2^ Institut Català de Paleoecologia Humana i Evolució Social (IPHES‐CERCA) Tarragona Spain; ^3^ Departament d'Història i Història de l'Art Universitat Rovira i Virgili Tarragona Spain; ^4^ Department of Biology University of Pisa Pisa Italy; ^5^ Department of Mechanical Engineering University College London London UK; ^6^ Department of Maxillo‐Facial Surgery and Plastic Surgery Necker – Enfants Malades University Hospital, Assistance Publique – Hôpitaux de Paris Paris France; ^7^ Department of Archaeology and Hull York Medical School University of York York UK; ^8^ Paleoanthropology, Senckenberg Centre for Human Evolution and Palaeoenvironment Institute for Archaeological Sciences, Eberhard Karls University of Tübingen Tübingen Germany

**Keywords:** endocast, human evolution, nasal cavity, paleoanthopology, paranasal sinusesr, R, segmentation, virtual anthropology

## Abstract

The use of non‐destructive approaches for digital acquisition (e.g. computerised tomography—CT) allows detailed qualitative and quantitative study of internal structures of skeletal material. Here, we present a new R‐based software tool, *Icex*, applicable to the study of the sizes and shapes of skeletal cavities and fossae in 3D digital images. Traditional methods of volume extraction involve the manual labelling (i.e. segmentation) of the areas of interest on each section of the image stack. This is time‐consuming, error‐prone and challenging to apply to complex cavities. *Icex* facilitates rapid quantification of such structures. We describe and detail its application to the isolation and calculation of volumes of various cranial cavities. The R tool is used here to automatically extract the orbital volumes, the paranasal sinuses, the nasal cavity and the upper oral volumes, based on the coordinates of 18 cranial anatomical points used to define their limits, from 3D cranial surface meshes obtained by segmenting CT scans. *Icex* includes an algorithm (*Icv*) for the calculation of volumes by defining a 3D convex hull of the extracted cavity. We demonstrate the use of *Icex* on an ontogenetic sample (0–19 years) of modern humans and on the fossil hominin crania Kabwe (Broken Hill) 1, Gibraltar (Forbes' Quarry) and Guattari 1. We also test the tool on three species of non‐human primates. In the modern human subsample, *Icex* allowed us to perform a preliminary analysis on the absolute and relative expansion of cranial sinuses and pneumatisations during growth. The performance of *Icex*, applied to diverse crania, shows the potential for an extensive evaluation of the developmental and/or evolutionary significance of hollow cranial structures. Furthermore, being open source, *Icex* is a fully customisable tool, easily applicable to other taxa and skeletal regions.

## Graphical abstract

*Icex* is a new R tool for the study of cranial cavities. It is tested here to automatically assess the size and shape of different cranial cavities on an ontogenetic sample of modern humans, three fossil hominins, and three species of non‐human primates. *Icex*'s performance shows the potential for extensive study on the development and/or evolution of hollow cranial structures. Being R‐based and open source, *Icex* is a fully customisable tool, easily applicable to other taxa and skeletal regions.

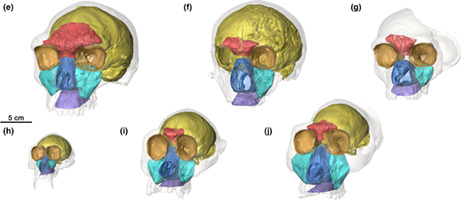

## 1 INTRODUCTION

The complex morphology of the cranium reflects the spatial and functional demands of the vital organs that it supports and the developmental and evolutionary constraints on morphogenesis (Bruner, 2018; Lieberman et al., 2000). Recent advances in technologies for digital imaging and virtual investigation (e.g. Profico et al., 2018; Spoor et al., 2000) allow highly detailed, non‐destructive characterisation of the form of the cranium and its internal structures using methods that are semi‐ or fully automated. In this paper, we present an advance in these approaches that facilitates the estimation and comparison of the volumes of cranial cavities and fossae together with an application of these methods to the study of growth changes in nasal, orbital, oral and frontal sinus volumes.

The human cranium encloses several organs (e.g. brain, sense organs) and serves diverse functions (e.g. air and food intake, first stage of digestion). These organs occupy space and fill cavities within the cranium; additionally, paranasal sinuses are anatomically and functionally associated with the nasal cavity (Keir, 2009). Other cavities or pneumatisations in the human cranium include the auditory canals, and the middle and inner ear (Singh et al., 2017). The study of some of these was possible in the past only by gross observation, and often after the destruction or dissection of the bone (Rae & Koppe, 2004). The availability of virtual methods has facilitated a number of studies examining the sizes, shapes, relationships, functions and evolutionary implications of such cranial cavities and fossae (de Oliveira et al., 2019; Evteev et al., 2014; Fatterpekar et al., 2008; Hill & Richtsmeier, 2008; Landi et al., 2021; Márquez, 2008; Márquez, Pagano, Lawson, et al., 2014; Noback et al., 2011; Profico et al., 2020; Zollikofer & Weissmann, 2008). In particular, the human neurocranial cavity has been intensively studied, since its morphology closely reflects that of the brain and it is straightforward to create a virtual endocast that closely mirrors its general form and some specific aspects of brain anatomy (e.g. Bastir, Godoy, et al., 2011; Bastir, Rosas, et al., 2011; Falk et al., 2005; Gunz et al., 2012; Kochiyama et al., 2018; Kranioti et al., 2011; Ogihara et al., 2018). Being non‐destructive, easily applicable and more versatile, this virtual approach has largely replaced the study of physical casts (e.g. Falk et al., 2000; Holloway, 2018).

Advances in virtual methods have also led to a flourishing of in‐depth studies of the lesser cavities of the cranium. The paranasal sinuses, the nasal cavity and the developmental and evolutionary interactions among these have been widely studied using traditional morphometric approaches based on linear measurements as well as geometric morphometric methods based on landmarks taken on digital images (e.g. Balzeau et al., 2017, 2022, 2021; Bastir & Rosas, 2013; Butaric et al., 2010, 2019; Holton et al., 2013; Maréchal & Heuzé, 2021; Prossinger et al., 2003; Rae & Koppe, 2004; Stansfield et al., 2021; Zollikofer et al., 2008). In these studies, the volumes of the paranasal sinuses were estimated either by digital (e.g. Balzeau et al., 2017; Noback et al., 2016) or physical means (e.g. by filling them with seeds, Márquez, Pagano, Delson, et al., 2014).

The increasing availability of large databases of digital anatomical images (CT/MRI), has facilitated large‐scale studies of form and variation (see for example Noback et al., 2011; Noback & Harvati, 2015; Shamaei‐Tousi et al., 2022) while increasingly, the functional consequences of variation in the form of cavities, particularly the nasal cavity and sinuses in living and fossil humans are investigated virtually using finite elements analysis (FEA) or computational fluid dynamics (CFD) (e.g. de Azevedo et al., 2017; Fitton et al., 2015; Godinho & O'Higgins, 2018; Inthavong et al., 2009; Wen et al., 2008; Wroe et al., 2018). Digital techniques have also been recently used to assess and visualise nasal pathologies in archaeological samples (Buzi et al., 2020; Mays et al., 2014). Beyond this, the development of methods for the study of the form of virtual representations of anatomy (Weber & Bookstein, 2011), has encouraged the investigation of anatomical variation of smaller cavities and fossae in the cranium. Recent studies have examined the auditory canals and the middle and inner ear (e.g. Bouchneb & Crevecoeur, 2009; Coleman & Boyer, 2012; Conde‐Valverde et al., 2018; Martínez et al., 2013; Uhl et al., 2016; Urciuoli et al., 2022) as well as the pneumatic cavities of the temporal bone (e.g. Balzeau & Grimaud‐Hervé, 2006; Balzeau & Radovčić, 2008; Han et al., 2007; Riga et al., 2022).

Several studies have assessed the volume of the oral cavity and the form of the soft tissues. Magnetic resonance tomography (MRT) allows detailed measurements to be taken from them (Iida‐Kondo et al., 2006). Novel methods for segmenting real‐time MRT data from the oral and nasal cavities have been applied to the assessment of function in speech (e.g. velum movements, Silva et al., 2012). Soft tissues are absent in fossil samples and material held in osteological collections, and so, in these cases estimation of these volumes is achieved through the use of skeletal measurements with appropriate adjustments for soft tissues (Jungers et al., 2003).

The most common approach to isolating a region of interest (ROI) and calculating its volume is through segmentation (Balzeau et al., 2010; Zollikofer et al., 1998). This consists of the selection or labelling of certain regions of images obtained by CT scans or magnetic resonance tomography (MRT; Weber, 2015). Such an approach may be applied in relation to skeletal samples of extant or fossil species and to soft and hard tissues in living humans (Pham et al., 2000). Segmentation can be entirely manual, with the ROIs selected by an operator. Manual segmentation is a demanding and time‐consuming process, requiring expertise (Balanoff et al., 2016, and references therein). It is recommended when the structure is complex or artefacts are already present, or when sediments infilling cavities, particularly in fossils, and the bone are of very similar radiodensity. At the same time, manual segmentation is inherently error‐prone (Huotilainen et al., 2014; Michikawa et al., 2017; Profico et al., 2018). In recent years, digital semi‐ and automatic methods have come to offer an alternative to manual segmentation, and this has facilitated the rapid production of virtual endocasts of improved accuracy. Segmentation methods are commonly applied directly to CT (or MRT) images (as does the software *xendocast*, Michikawa et al., 2017). Alternatively, volumes can be calculated directly from 3D surface meshes—derived from prior segmentation and 3D reconstruction—using recently developed software tools (*endex*, Beaudet & Gilissen, 2018; *endomaker*, Profico et al., 2020). While *xendocast* and *endex* are designed specifically to derive brain endocasts, *endomaker* is an endocast‐focused implementation of previous digital tools (specifically, *CA‐LSE* and *AST‐3D*, Profico et al., 2018), developed in R to be applicable to a wide range of skeletal cavities and fossae.

In this study, we present a new open‐source tool (*Icex*—*Internal Cavity EXtraction* tool), developed in R and based on *endomaker*, *CA‐LSE* and *AST‐3D*. *Icex* can isolate and extract the surface meshes of diverse cranial cavities and fossae from an entire cranial mesh, using a landmark configuration to fully automate the process. Using *Icex*, the cavity of interest (COI) is rapidly extracted and its volume is calculated based on the smallest polygon delimitating the cavity or fossa (the α‐shape, Edelsbrunner & Mücke, 1994; Lafarge et al., 2014). The mesh can also be exported and edited, and the calculation of the α‐shape can then be undertaken separately, using a different function (*Icv—Internal Cavity Volume* tool). Here, we present and assess the performance of *Icex*, by segmenting and computing the volumes of the nasal cavity (NC), maxillary sinuses (MS), orbits (OR), upper oral cavity (palate, PA) and frontal sinuses (FS), from a sample of modern humans of different developmental stages. In addition, we test *Icex* on three human fossils: one is the remarkably well‐preserved fossil hominin Kabwe 1 (also known as Broken Hill 1, often designated as *Homo heidelbergensis s.l*. but see discussion in, e.g. Harvati & Reyes‐Centeno, 2022), and two are attributed to the species *Homo neanderthalensis*, namely Gibraltar 1 (also known as Forbes' Quarry) and Guattari 1 (previously known as Circeo 1). Finally, we test *Icex* on a subsample of sixnon‐human primates. In so doing, we demonstrate the wide application of this toolkit to the study of ontogenetic and evolutionary variation in cranial cavity volumes.

## 2 MATERIALS AND METHODS

### 2.1 Cranial 3D models

The sample of modern humans consisted of 10 individuals of known age, ranging from newborn to adult (defined as such based on the eruption of M3). These are from two different databases: the CT‐scan collection from Necker—Enfants Malades University Hospital in Paris (Galiay et al., 2022) and the New Mexico Decedent Image Database (Edgar et al., 2020, https://nmdid.unm.edu). The 3D models of Kabwe 1, Gibraltar 1 and Guattari 1 were downloaded from the NESPOS Database (available as a digital archive: https://archiv.neanderthal.de/data/). Two specimens of both sexes for each of the species *Pan troglodytes*, *Gorilla* and *Macaca sinica*, came from the National Museum of Natural History (Washington, USA) and were obtained as surface meshes from the ‘Smithsonian Open Access’ database (available at https://www.morphosource.org/). The complete specimen list is reported in Table S1, in Supporting Information. All modern human data were anonymised, and the ethical approvals were authorised by the corresponding institutional ethical committees. Cranial 3D surface meshes were generated by reconstructing the CT images in Avizo Lite software (FEI V9.2., Thermo Fisher Scientific).

### 2.2 Landmark configuration

**FIGURE 1:**
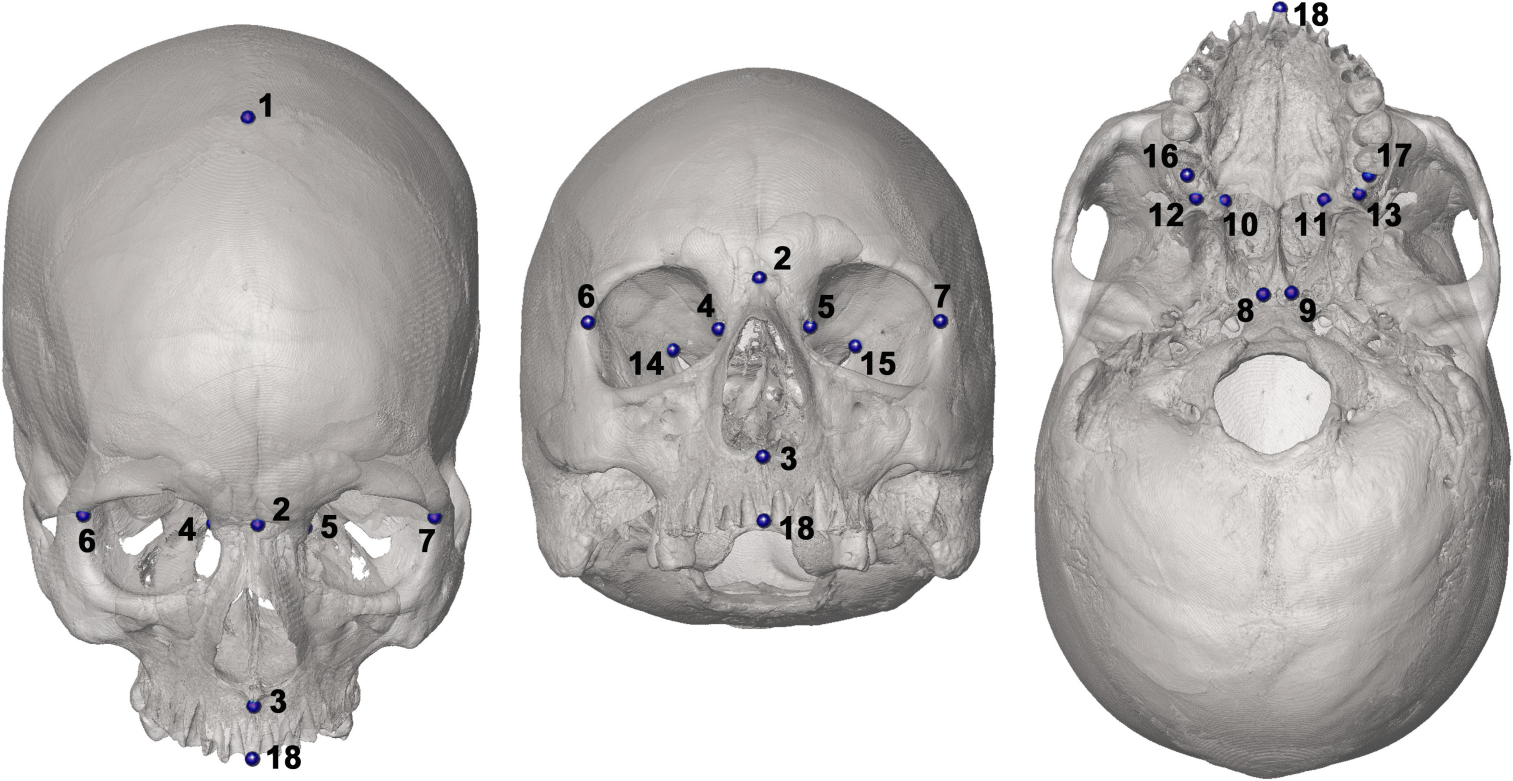
Full landmark configuration. The landmarks are numbered according to the order in which they were taken (see Table 1).

We defined a landmark (LM) configuration comprising 18 cranial anatomical points (Figure 1) to extract all of the COIs for this study (NC, MS, OR, PA, FS). Alternatively, the user can define a specific LM configuration to extract a single COI (see Figures S1–S3 in Supporting Information). The full configuration used in this study is shown in Figure 1 and the LMs are reported in Table 1, along with an indication of the COI for which they are sampled. A detailed description of the parameters used in *Icex* is included in Supporting Information 1 and Table S2 in Supporting Information.

**TABLE 1:** List of the landmarks forming the full configuration, reported in the order of sampling. The cavities for which they are used are reported (NC, nasal cavity; MS, maxillary sinuses; OR, orbits; PA, palate; FS, frontal sinuses). Nomenclature and descriptions for the points 1–7 and 18 are sourced from White and Folkens (2000), except for 3*, which is sourced from Caple and Stephan (2016).

Number	Name	Description	Cavities				
			NC	MS	OR	PA	FS
1	Bregma	Point of intersection between the coronal and sagittal sutures	✓	✓			
2	Nasion	Midline point of intersection between the two nasal bones and the frontal bone	✓				
3*	Subspinale	Deepest midline point below the anterior nasal spine	✓				✓
4	Lacrimale R	Point of intersection between the posterior lacrimal crest and the frontolacrimal suture (right side)	✓	✓	✓		✓
5	Lacrimale L	Point of intersection between the posterior lacrimal crest and the frontolacrimal suture (left side)	✓	✓	✓		✓
6	Frontomalare orbitale R	Point of intersection between the frontozygomatic suture and the inner orbital rim (right side)		✓	✓		✓
7	Frontomalare orbitale L	Point of intersection between the frontozygomatic suture and the inner orbital rim (left side)		✓	✓		✓
8	—	Tip of the right wing of the vomer	✓	✓			✓
9	—	Tip of the left wing of the vomer	✓	✓			
10	—	Posterolateral corner of the hard palate R	✓	✓			
11	—	Posterolateral corner of the hard palate L	✓	✓			
12	—	Posteroinferior margin of maxillopalatine suture (right side)	✓	✓			
13	—	Posteroinferior margin of maxillopalatine suture (left side)	✓	✓			
14	—	Superior orbital fissure (right side)			✓		
15	—	Superior orbital fissure (left side)			✓		
16	—	Posterior margin of last maxillary molar alveolus (right side)				✓	
17	—	Posterior margin of last maxillary molar alveolus (left side)				✓	
18	Prosthion	Anteriormost midline point of the maxillary alveolar process				✓	

### 2.3 The protocol

**FIGURE 2:**
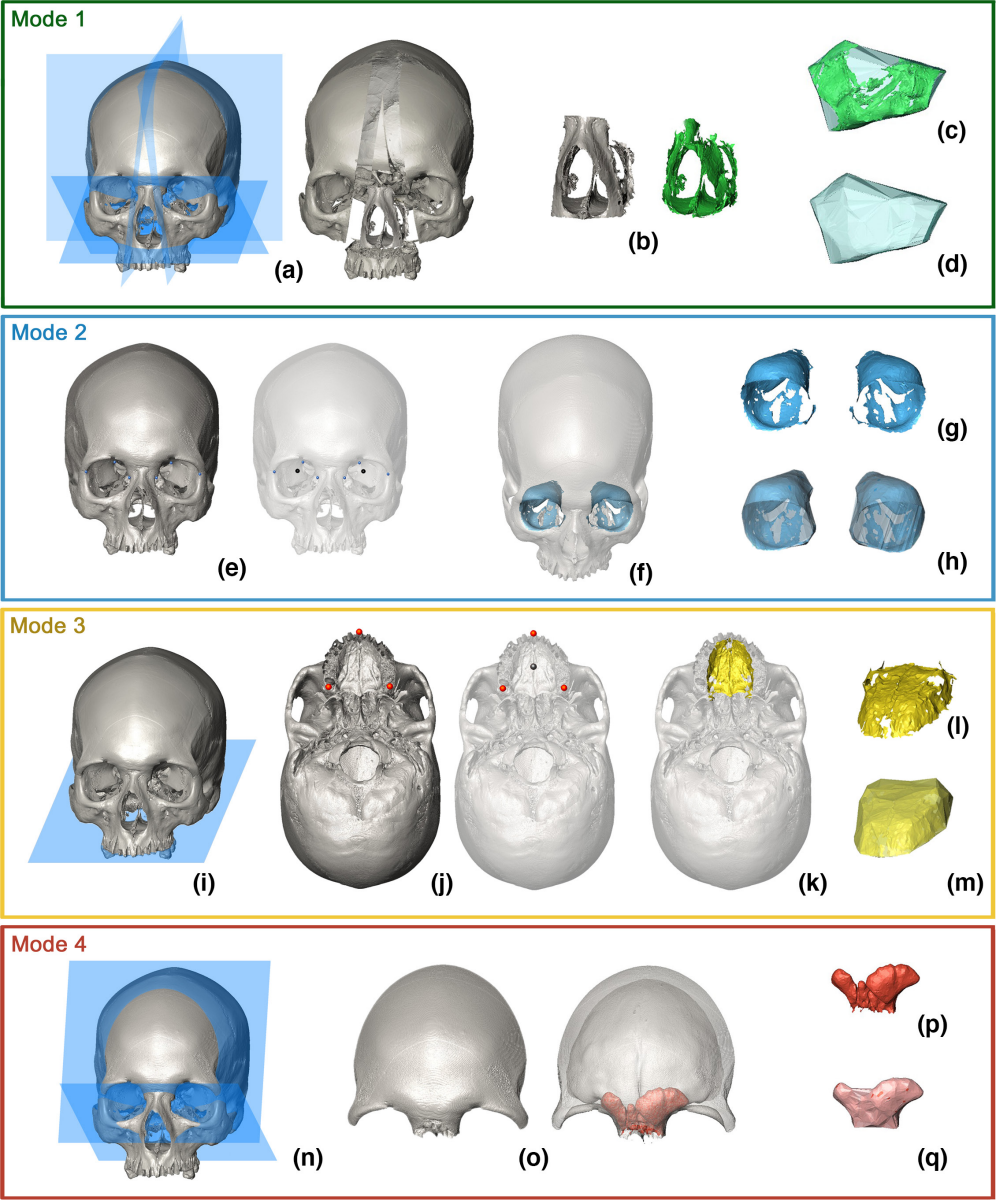
The different modes of *Icex*, with sample cavities: Mode 1 (nasal cavity), Mode 2 (orbits), Mode 3 (palate) and Mode 4 (frontal sinuses). The single passages are explained in the text.

The inputs to *Icex* comprise a 3D mesh (e.g. .ply, .stl, .obj format files) and the landmark configuration (i.e. matrix of 3D coordinates). To speed up and simplify computation, the meshes are downsampled to 500,000 triangles at the beginning of the process (see the full script in Supporting Information 1). *Icex* allows the selection of one of four alternative Modes for the extraction of COIs, depending on the COI to be extracted. In general, *Icex* can include the following steps: (i) cutting of the mesh; (ii) automatic selection and extraction of the COI; (iii) creation of the α‐shape and calculation of the volume and (iv) exporting of the meshes of the COI and its α‐shape (see Figure 2). The exact steps used depend on the choice of the Mode, as explained below. The choice of the Mode depends on the cavity or fossa to be measured. Steps ii, iii and iv are included in all four Modes. The *mode* selection is an argument of the function, while step (iii) creation of the α‐shape and calculation of the volume, can be performed separately, using the function *Icv* on its own, for example, applied to the mesh of a COI generated using alternative software.

#### 2.3.1 Mode 1

Mode 1 (Figure 2a–d) can be used for cavities such as the nasal cavity and maxillary sinuses. It requires the isolation of the COI by cutting of the surface mesh (Figure 2a). This is performed by the function *cutMeshPlane* (package *Morpho*, Schlager, 2017). This function requires the definition of cutting planes using sets of three non‐collinear landmarks (see Figure 1 and Table 1). The argument ‘*planes*’ sets the cutting plane and the argument ‘*keep*’ defines which part of the mesh is kept after the cut. This way, the skeletal portion of interest (which houses the COI) is isolated and suitable as input for *endomaker* (package *Arothron*, Profico et al., 2021; Figure 2b). Lastly, the function *Icv* builds the α‐shape around the extracted inner surface (Figure 2c,d); more details are given in subsection 2.4, below. In the case of nasal cavity, 5 planes are used to cut the cranial mesh, while for each of the maxillary sinuses, 6 planes are required.

#### 2.3.2 Mode 2

Mode 2 (Figure 2e–h) can be used for each of the orbits. It can also be applied to the upper oral volume when teeth are absent (e.g. in young or otherwise edentulous individuals), otherwise Mode 3 is suggested. Mode 2 does not include the cutting of the mesh; thus, no planes are required and the argument ‘*planes*’ and ‘*keep*’ are set to NULL. Mode 2 calculates the arithmetic mean of three landmarks to define the barycentre of COI (Figure 2e). The barycentre is subsequently defined as a single point of view (POV) for the application of *AST‐3D* (Figure 2f, see Profico et al., 2018). *Icv* (Figure 2f) is then applied to the inner surface of each orbit (Figure 2g).

#### 2.3.3 Mode 3

Mode 3 (Figure 2i–m) is a variant of Mode 2, applicable to, for example, the upper oral volume if the palatal surface alone adequately defines the COI. Removal of the upper teeth is the first step, requiring the setting of a single plane defining the alveolar limit (Figure 2i) followed by cutting at the level of this plane. The function then proceeds as in Mode 2: identification of the barycentre as the arithmetic mean of three landmarks (Figure 2j) and its use as the POV for the application of *AST‐3D* (Figure 2k). The inner surface is extracted (Figure 2l) and the COI volume is calculated by *Icv* (Figure 2m).

#### 2.3.4 Mode 4

Mode 4 (Figure 2n–q) can be applied to frontal sinuses. Its application requires the preliminary cutting of the cranium by two planes (Figure 2n) to isolate the frontal bone, to which *CA‐LSE* (Profico et al., 2018) is subsequently applied to extract the frontal sinuses (Figure 2o). *Icv* is then applied to the extracted surface (Figure 2p) and the volume of the COI is calculated (Figure 2q). In infants, the reduced frontal sinuses are often composed of several distinct pneumatised cells. Mode 4 accommodates this, by allowing the operator to decide if volumes are calculated from the biggest cell (*multiple* = FALSE) or all of them (*multiple* = TRUE). In the latter case, it is recommended to apply *Icv* to each cell forming the sinus, separately.

### 2.4 Calculation of volume and saving of meshes

Once the COI is extracted, using one of the four Modes, the function *Icv*, embedded in *Icex*, builds the α‐shape around the COI. The setting of the argument *alpha* (see Table S2 in Supporting Information) determines the closeness of fit of the α‐shape to the COI (Figure 2d,h,m,q; Figure S4 in Supporting Information). The volume is then calculated based on the empty voxels included in the α‐shape (i.e. voxels not containing any vertex from the surrounding mesh, Profico et al., 2020). So, the estimate of volume is affected by the correct building and the closeness of fit of the bounding polygon to the actual shape, which can be regulated by setting the *alpha* argument (Figure S4 in Supporting Information). After the computation of the α‐shape, *Icex* provides a comprehensive visualisation of the COI, the α‐shape and the two combined (as seen in Figure S4a–c), to assess the quality of the extraction. At this point, using the R console the user can decide whether to export the two meshes (COI and α‐shape). In case of affirmative response (‘y’), the two meshes are saved in the working folder, otherwise (‘n’) the protocol is terminated. The output includes the volume of the COI. *Icv* can be separately applied if the extracted COI needs to undergo editing using different imaging software. This can be done by simply exporting the extracted COI, processing it, and using the function *Icv* instead of *Icex* on the reloaded mesh.

## 3 RESULTS

**FIGURE 3:**
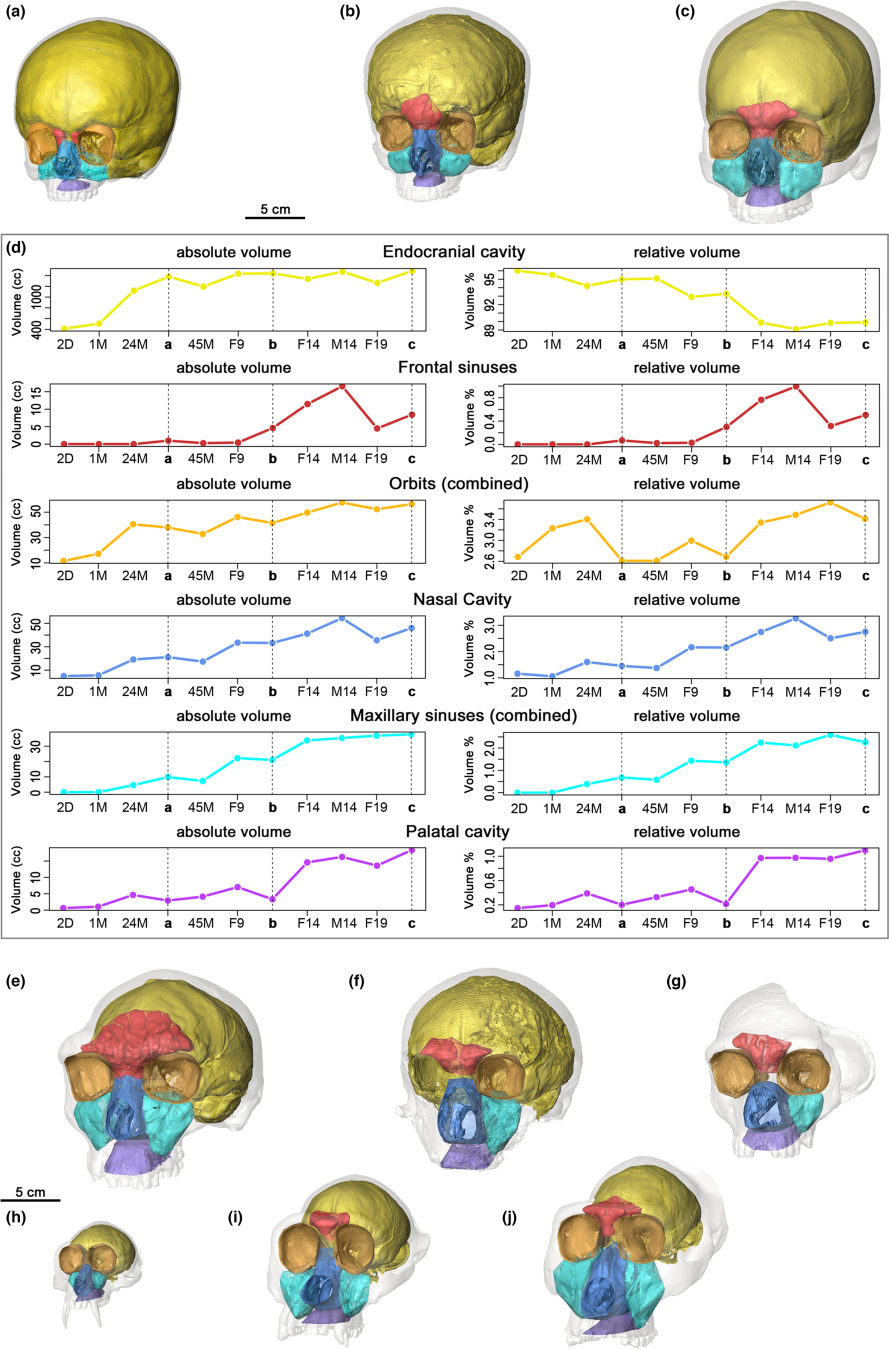
Visualisation of the cavities extracted in nine individuals of the sample. (a) sub‐adult female, 35 months; (b) sub‐adult male, 9 years; (c) adult male, 19 years. (d) absolute and relative volumes for each cavity in the modern human sample. 2D: 2 days; 1 M: 1 month; 24 M: 24 months; 45 M: 45 months; 9, 14, 19: 9, 14 and 19 years, males (M) and females (F). Fossil human subsample: Kabwe 1 (e); Guattari 1 (f); Gibraltar 1 (g). Primate subsample: *Macaca sinica*, male (h); *Pan troglodytes*, male (i); *Gorilla gorilla*, female (j).

A detailed summary of the various input parameters used in *Icex* to calculate volumes of the COIs examined here is included in Supporting Information 1. Table 2 summarises the absolute volumes measured across all crania in our sample. Figure 3a–d highlights the changes in volume of the COIs during ontogeny. Figure 3d plots against age the absolute (cm^3^) and relative (%) volumes for each cavity extracted by *Icex*, including the endocranial cavity, extracted by *endomaker* (Profico et al., 2020). For the orbits, the maxillary sinuses and frontal sinus (where separate) the total volume of both sides is plotted. A detailed representation in different norms of all cavities and the respective α‐shapes of one individual (male, 19 years old) is presented in Figure S5 in Supporting Information, along with the nasal cavity and left maxillary sinus (complete with the α‐shapes) of Guattari 1, as an example of the application to a fossil specimen.

**TABLE 2:** Absolute volumes (cm^*3*^) of each cavity in the whole sample. NC: nasal cavity; MS‐l, left maxillary sinus; OR‐l, left orbit; OR‐r, right orbit; PA, palatal cavity; FS, frontal sinuses; EC, endocranial cavity. (M), probably male; (F), probably female; na, not available. The species of non‐human primates are reported in the individuals' names as Pa_tr_ (*Pan troglodytes*), Go_go_ (*Gorilla gorilla*), Ma_si_ (*Macaca sinica*). * The endocranial volume of Guattari 1 is taken from Holloway et al., 2004.

Individual	Age	Sex	NC	MS‐l	MS‐r	OR‐l	OR‐r	PA	FS	EC
2DF	2 days	F	4.97	na	na	5.73	5.77	0.63	na	411.45
1M11DF	1 month, 11 days	F	5.63	na	na	8.53	8.62	1.04	na	507.31
24M21DF	24 months, 21 days	F	19.12	2.17	2.42	20.13	20.29	4.63	na	1119.49
35M24DF	35 months, 24 days	F	21.13	5.06	4.8	18.86	19.05	2.93	0.99	1379.66
45M7DF	45 months, 7 days	F	17.31	3.56	3.74	16.58	16.22	4.12	0.28	1195.09
F9_127657	9 years	F	33.51	11.08	11.13	23.03	23.17	7.04	0.38	1434.16
M9_195011	9 years	M	33.26	10.78	10.23	20.43	21	3.32	4.61	1439.05
F14_104011	14 years	F	41.22	16.31	17.44	24.94	24.73	14.6	11.47	1337.21
M14_136300	14 years	M	54.44	17.89	17.43	29.13	28.5	16.27	16.57	1473.22
F19_115804	19 years	F	35.53	18.61	18.23	25.71	26.6	13.6	4.48	1262.77
M19_106561	19 years	M	45.99	19.03	18.71	28.12	28.26	18.38	8.43	1486.6
Kabwe 1	Adult	(M)	75.5	35.53	32.63	47.47	47.88	31.42	38.78	1270.35
Gibraltar 1	Adult	(F)	55.49	21.14	na	37.22	36.88	18.73	7.50	na
Guattari 1	Adult	(M)	96.95	38.89	na	40.45	na	17.07	8.40	1380*
Pa_tr_220062	Adult	F	55.10	14.00	14.70	29.17	29.28	11.54	3.93	411.09
Pa_tr_174704	Adult	M	56.45	13.92	14.75	29.18	29.22	14.57	4.23	410.76
Go_go_590951	Adult	F	88.65	31.19	40.68	30.25	30.39	22.82	9.29	396.29
Go_go_174712	Adult	M	147.10	56.92	65.40	41.24	43.20	21.28	14.28	487.91
Ma_si_271190	Adult	F	4.33	1.03	0.98	6.68	6.61	1.57	na	60.32
Ma_si_15259	Adult	M	7.46	1.95	1.37	9.26	9.18	3.15	na	86.40

To test for inter and intra‐observer error in the calculation of the endocranial volumes, the landmark configurations of the modern human individuals older than 2 years old (i.e. with detectable maxillary sinuses) were acquired three times by two operators. A Multivariate ANOVA was performed. Results show significant differences among individuals (*p*‐value < 0.001; *R* ^2^ = 0.997) and no significant differences among replicates (*p*‐value = 0.991; *R* ^2^ = 0.0002).

The volumes calculated using *Icex* highlight that, limiting the observation to our sample, in modern humans there is a rapid increase in the endocranial volume (range = 411 cm^3^–1487 cm^3^) from birth to 2 years of age, reflecting brain growth. Beyond this age, the rate of endocranial volume growth slows dramatically. Considering the growth of endocranial volume as a proportion of all measured volumes, we observed a gradual reduction beyond 2 years of age (from 96.0% to 89.1%) reflecting differences in timings and rates of expansions of cavities. The frontal sinus volume ranged between 0 and 17 cm^3^ and, in our sample, seems to drastically increase after year 9. The relative contribution of the frontal sinuses to total cavity volume was minimal (from 0% to 1.0% in adults). The orbital volume increased more from birth to 2 years of age (from 11.5 cm^3^ to 40.4 cm^3^) than over all subsequent age intervals (from approximately 38 cm^3^ to 57 cm^3^). The relative contribution of orbital volume remained rather constant during the entire growth period, varying between 2.6% and 3.7%. At birth, the nasal cavity volume is approximately 5 cm^3^ and it gradually increases during growth, to approximately 54 cm^3^ in adults. The same trend was observed in the relative volume of the nasal cavity, that is, 1.1% at birth and 3.3% at adulthood. Maxillary sinuses show a similar positive trend of volume change during growth (from 0 to 38 cm^3^). Their relative contribution constantly increases until adulthood (from 0% to 2.6%). The upper oral volume shows a continuous gradual increase during growth (from approximately 1 to 18 cm^3^).

The fossil subsample allowed us to show the potential of *Icex* when applied to damaged specimens. Based on Table 2 it is possible to compare the volumes of COIs in fossils with those of the adult modern humans in our sample. The two Neanderthals show missing and/or not preserved portions of the COIs; though, the anatomical boundaries delimiting some of these are well enough preserved for the tool to identify them and isolate their general structures. The volume of NC reaches its maximum value in Guattari 1, with Kabwe 1 and Gibraltar 1 having lower volumes, although all three have larger NC volumes than the modern humans in our sample. Given the lack of most of the boundaries (Guattari 1) and sediment infilling (Gibraltar 1), it was not possible to calculate the volume of the right MS among the fossils, except for Kabwe 1. The left MSs, on the other hand, show notably higher values compared to modern humans. Indeed, that of Guattari 1 is nearly double that of the adult modern human male of our sample. PA and FS in the Neanderthals have values similar to those of modern humans, while in Kabwe both volumes are consistently larger. Lastly, the volumes of the orbits seem to be larger in fossils than modern humans, with the largest ORs recorded, again, in Kabwe 1.

*Icex* was also tested on a primate subsample. To attempt the extraction on specimens of different sizes, a female and a male individual of both great apes (species *Pan troglodytes* and *Gorilla gorilla*) were included, as well as macaques (*Macaca sinica*). To extract COIs from such a diverse sample, some adjustments were made to both parameters and the landmark configuration, but all, when present, were successfully extracted. As expected, the interspecific size difference is reflected in the subsample, with the gorillas displaying larger volumes for all COIs. Among great apes, the orbits seem to be least variable in size, with a small range of volumes in *Pan* and *Gorilla*, while the other COIs show more marked differences in volume (e.g. NC, MSs, FSs). In our limited sample, cavities seem to vary less in volume between the sexes in *Pan* than in *Gorilla* and *Macaca*. In both the latter species, MS volumes are also somewhat larger in the male.

## 4 DISCUSSION

*Icex* enables the extraction of cavities and the estimation of their volumes from tomographic scans of bones. Here it was applied to a case study concerning changes with age in the volumes of cranial cavities. The software allowed us to gather cavity and fossa volume data from a series of crania in a fast, convenient and reproducible way. Algorithms already validated in previous works, by us and other authors (*ashape3d*, Lafarge et al., 2014; *CA‐LSE* and *AST‐3D*, Profico et al., 2018; *endomaker*, Profico et al., 2020) were combined into a single function that can be adapted to different skeletal elements. The visual output (COI + α‐shape) allows the user to adjust the parameters according to the aims of their study and to repeat the process in a time‐efficient fashion. We tested the use of *Icex* in a number of individuals at different ages and this highlighted its robustness in identifying sets of COI‐specific parameters. By using these parameters, the software was applicable to the whole human and primate sample, with only a few adjustments needed, as explained below.

In studying a different human species (e.g. *Homo heidelbergensis*, *Homo neanderthalensis*), we recommend slight changes in the LM configuration, since cranial morphology can vary considerably between different species. This is particularly true for facial morphology, which in modern humans diverges significantly from general aspects of morphology shared by archaic human species (Lacruz et al., 2019). This translates into changes in, for example, the anterior aspects of the maxillary sinuses and in their spatial relationship with the nasal cavity. With *Icex* it was possible to efficiently extract all the cavities in the fossil subsample with minimal adjustments to overcome the issues presented by differences in facial morphology. The wider interorbital area of the fossils required adjustment of the lacrimal landmarks (i.e. 4 and 5, Table 1) placing them more anteriorly. This was to obtain a more accurate intercept at the boundaries of the nasal cavity and maxillary sinuses. Secondarily, the larger size of the nasal and paranasal structures required different values of *param2* (NC) and *param1* (MSs), for these COIs only (see Table S2 in Supporting Information). These modifications ensured COIs were adequately extracted, but they did not affect the estimation of the volumes themselves.

Apart from these changes, due to species‐specific morphological differences, variation among modern humans was efficiently accommodated by *Icex* without major changes in the input parameters. Another exception concerns the frontal sinuses in some juvenile specimens and the two Neanderthals. In the first case, the small, complex structure of the incipient frontal sinus in three juvenile specimens (namely, 35M24DF, 45M7DF and F9_127657, see Table 2) required, as stated in the Methods Section (2.3.4 Mode 4), a preliminary extraction by setting the argument *multiple* to TRUE, with subsequent removal of frontal cavities that are not part of the FSs by using imaging software (i.e. Avizo), with subsequent, separate application of *Icv* to the two meshes. The volume was then calculated by summing the volumes obtained from *Icv*. In both the two Neanderthals, FS is formed by two separated cavities, albeit with partial overlap. Also in this case, the argument *multiple* was set to TRUE to keep both portions and the mesh of the FSs was ‘cleaned’ using imaging software to remove the portions that were not part of the FSs, before applying *Icv*.

An additional assessment of the extraction of FSs was possible because the fossils included in this study were also sampled in a recent study of these cavities by Balzeau et al. (2022). In that study, the extraction was performed by manual segmentation, and the volumes calculated do not differ much from the results we obtained. The difference is due to the definition of boundaries of the sinuses themselves: for example, the inferior extension, which directly connects with the ethmoid pneumatisation is not always well‐defined and/or preserved (Balzeau et al., 2021). We defined the boundaries of the FSs by cutting the cranium along planes constructed using homologous external points, whereas manual segmentation defines the boundaries based on the expertise of the operator. In general, it should be noted that the very short time needed for the actual extraction and measurement of COIs by *Icex* can ‘compensate’ for the time lost in editing volumes extracted from individuals who present problematic aspects of sinus anatomy.

The poorer preservation of the two Neanderthal individuals (compared Kabwe 1), did not allow the extraction of some COIs either because they are missing (i.e. right orbit of Guattari 1) or lack a considerable portion of their boundaries (i.e. right maxillary sinus of Guattari 1). In one case, the COI was filled with sediment (i.e. right maxillary sinus of Gibraltar 1). However, for extraction of PA and NC, even though damaged and poorly preserved, the general boundaries were identified, and the volumes could be calculated. In these fossils, our results suggest that mid‐facial prognathism in Neanderthals is paired by a large NC volume, with MSs and PA volumes seemingly contributing less. In Kabwe 1 all cavities reflect it large size, except for the relatively low endocranial volume, which was estimated by reflected relabelling (Gunz et al., 2009). These observations are very limited but point to the potential application of *Icex* in comparative studies of cranial cavities in fossils.

The versatility of *Icex* has also been demonstrated using the primate subsample. These crania vary considerably in size and shape. In general, the parameters used for the humans worked as well for the primates, with some adjustments, for example, the value of *param1* increased to better extract the massive NC of the male gorilla (from 1 to 1.5), or the more elongated MSs in the great apes (from 0.1 to 0.8), or to better intercept the proportions of the ORs in primates (from 0.2 to 0.5), which are slightly more elongated than in humans. Anatomical differences may require alterations of the landmark configuration. Thus, in the applications to primates, as well as in studies of species with strong prognathism, or a snout, the relative positions of landmarks (e.g. *prosthion* and the posterior margins of the molar alveoli), as well as their presence (e.g. *subspinale*) can vary. In this study, the extraction of COIs affected by such variations was still possible, for example, by making *subspinale* coincide with the *prosthion*. In the case of PA of certain species of primates, it is necessary to consider the slight upward curvature of the maxillae (i.e. airorhynchy): this was accomplished in our analysis, but adjustments of the landmark configuration used in extracting PA may be required to perform the same analysis on a more diverse primate sample (e.g. comprising orangutans). In our limited sample, variation in volumes seems to be influenced by the degree of sexual dimorphism, although some cavities seem to be less dimorphic. Some cavities seem to vary more than others among the great apes (including humans). While these findings are based on a limited sample, they point to the potential benefits of *Icex* in facilitating future studies of this kind using larger samples.

In conclusion, *Icex* allowed us to assess and compare growth changes in the volumes of different cranial cavities. The calculation of volume using the α‐shape presents several advantages: (i) it overcomes the incompleteness of the structures caused by the stage of development (e.g. in newborns or subadults) or poor skeletal preservation (e.g. in archaeological material); (ii) it limits the influence of possible artefacts present in the COIs by delimiting them with reproducible boundaries and (iii) it offers avenues by which the volumes of complex cellular structures such as sinuses can be estimated.

The limited sample we analysed shows that *Icex* offers the possibility of carrying out extensive studies on the development or evolution of cranial pneumatisation. These, as the flourishing of recent studies demonstrates, may be of great interest in describing evolutionary transformations (e.g. MSs and FSs in the evolution of facial morphology; endocast in palaeoneurology) and adaptations (e.g. NC and adaptation to climate; inner and middle ear and the evolution of speech). *Icex* represents a fast and repeatable alternative to manual segmentation that can facilitate and speed up such analyses. Our limited findings using small samples point to interesting similarities and differences in how the volumes of cranial cavities develop, perhaps accommodating the growth and development of adjacent elements. This can be further investigated using larger data sets. The software we present will facilitate such future studies and has wide applicability to different skeletal elements and species.

## AUTHORS' CONTRIBUTIONS

CB and KH conceived the study; CB, CL and RHK collected and curated data; CB, AP, CL, POH and MM designed the statistical analysis; CB, AP and CL performed the analysis; CB and AP wrote the R code; CB and AP drafted the first version of the manuscript; and all authors reviewed the manuscript and approved the paper.

## Supporting information


Data S1.

## Data Availability

The full R code for *Icex* and example data are available on Zenodo: https://zenodo.org/record/6642828.
