# Supplementary material for: Icex: Advances in the automatic extraction and volume calculation of cranial cavities
Source: J Anat. 2023 Feb 11;242(6):1172–83. doi: 10.1111/joa.13843 (PMC10184549; doi:10.1111/joa.13843)
Supplement: Supplementary file 1 — Data S1. [file JOA-242-1172-s001.pdf]

Supporting information:

1 – Full script with runnable example

Figs S1-S3: separate landmarks configs for COI

Fig S4: building of the α-shape and setting of *alpha*

Table S1: sample used

Table S2: arguments of the function *Icex*

Supporting Information 1 – Example code for *Icex* and *Icv*

Cavity of interest (COI)-specific values for each argument of *Icex* are here presented. Some arguments identify the individual (*sur, set*), others are determined by the Mode applied (*planes, keep, multiple*), while *param1, param2* and *alpha* can be changed to obtain an optimal extraction of the COI and calculation of its volume. For these, in testing the function, we tried to identify values that would work on a heterogeneous sample as the one we used. *param1* and *param2* were needed to be changed only between the COIs, and not between individuals, with few exceptions (see section 4, Discussion in the Main Text). The value of the *alpha* argument has been set to 20 for all sample and all cavities, except for the endocast (EC) and palate (PA). In the first case, *alpha* has a default volume in *endomaker*, in the second case, the value has been set to 50. This is because a smaller polygon enveloping the shape (i.e., strictly adhering to the inner surface of the palate) would not effectively approximate the overall dimension the upper oral cavity. The higher value of *alpha*, in this case, allows for the isolation of a larger portion of the cavity better approximating the inner volume of the upper oral cavity.

Below we provide the code for *Icex* and its application for the extraction and volume calculation of each COI. We also provide a version of the code including the parameters for the application of the COI-specific partial landmark configurations. Lastly, we provide an example for the application of *Icv* only.

Installation of the Arothron package and importing of example data provided:

```
install.packages("Arothron")

library(Arothron)

load("IcexData.rda")
```

Installation of the other packages needed:

```
install.packages("Morpho")

install.packages("alphashape3d")

install.packages("rgl")

install.packages("Rvcg")

library(Morpho)

library(alphashape3d)
```

```
library(rgl)

library(Rvcg)
```

It is possible to upload own meshes (e.g., .ply files) by installing the package Morpho and using the function for reading 3d meshes. We also applied a decimation to 500k triangles:

```
sur<- vcgQEdecim(ply2mesh("name_mesh.ply"),500000)

set=read.table("name_set",sep=";",dec=".",col.names = NA)
```

In this case the first two arguments of the function will be `sur` and `set`.

Load the functions provided in the supporting code available on Zenodo (https://zenodo.org/record/6642828). On Zenodo we supplied example data for `sur` and `set`.

After the visualization of the different COIs, by running *Icex* as explained below for each single cavity, it is possible to automatically save the meshes of both the extracted cavity and the α-shape in the working folder by typing on the R console the affirmative response ("y"), otherwise it can be typed a negative response ("n") prior to restart the process.

Extraction of the Nasal Cavity (NC). The parameters suggested are those used in our study:

```
NC<-Icex(sur=sur, set=set,
planes=list(c(1,8,9),c(3,10,11),c(1,4,12),c(1,5,13),c(2,8,9)),keep=c(TRUE,FALSE,
TRUE,FALSE,TRUE), param1=1, param2=4, alpha=20, mode=1)
```

To print the volume of the cavity, type:

```
NC$volume
```

Or simply run:

```
NC
```

to see all the attributes of the object (volume included).

Extraction of the Left Maxillary Sinus (MS_l) and/or the **Right Maxillary Sinus (MS_r)**. The parameters suggested are those used in our study:

```
MS_l<-Icex(sur=sur, set=set,
planes=list(c(4,5,8),c(1,8,9),c(1,6,12),c(1,7,13),c(4,9,11),c(1,5,11)),
keep=c(FALSE,TRUE,TRUE,FALSE,FALSE,TRUE), param1=0.1, param2=0.4, alpha=100,
mode=1)

MS_r<-Icex(sur=sur, set=set,
planes=list(c(4,5,8),c(1,8,9),c(1,6,12),c(1,7,13),c(5,8,10),c(1,4,10)),
keep=c(FALSE,TRUE,TRUE,FALSE,TRUE,FALSE), param1=0.1, param2=0.4, alpha=100,
mode=1)
```

To print the volume of the cavity, type:

```
MS_l$volume

MS_r$volume
```

Or simply run:

```
MS_l

MS_r
```

to see all the attributes of the object (volume included).

A reduced landmark configuration limited to the first 13 landmarks (see Figure S1 in Supporting Information Figures) can be applied without changing the parameters set for *planes* and *keep*, if interested in NC and MSs only.

Extraction of the Left Orbit (OR_l) and/or the **Right Orbit (OR_r)**. The parameters suggested are those used in our study:

```
OR_l<-Icex(sur=sur,set=set, planes=NULL, keep=NULL, param1 = 0.2, param2=NULL, alpha=20, center=c(5,7,15), mode=2)

OR_r<-Icex(sur=sur, set=set, planes=NULL, keep=NULL,param1 = 0.2, param2=NULL, alpha=20, center=c(4,6,14), mode=2)
```

It can be applied a reduced landmark configuration (see Figure S2, a in Supporting Information Figures) for the extraction of the orbits only, by changing parameters as follows:

```
#OR_l<-Icex(sur=sur,set= "subset", planes=NULL, keep=NULL, param1 = 0.2, param2=NULL, alpha=20, center=c(3,4,6), mode=2)

#OR_r<-Icex(sur=sur, set= "subset", planes=NULL, keep=NULL,param1 = 0.2, param2=NULL, alpha=20, center=c(1,2,5), mode=2)
```

To print the volume of the cavity, type:

```
OR_l$volume

OR_r$volume
```

Or simply run:

```
OR_l

OR_r
```

to see all the attributes of the object (volume included).

Extraction of the Upper Oral Cavity (or ‘palate’, PA). The parameters suggested are those used in our study:

```
PA<-Icex(sur=sur, set=set, planes=list(c(16,17,18)), keep=FALSE, param1=0.5, param2=NULL, alpha=50, center=c(16,17,18), mode=3)
```

It can be applied a reduced landmark configuration (see Figure S2, a in Supporting Information Figures) for the extraction of the orbits only, by changing parameters as follows:

```
PA<-Icex(sur=sur, set="subset", planes=list(c(1,2,3)), keep=FALSE, param1=0.5, param2=NULL, alpha=50, center=c(1,2,3), mode=3)
```

To print the volume of the cavity, type:

```
PA$volume
```

Or simply run:

```
PA
```

Extraction of the Frontal Sinuses (FS). The parameters suggested are those used in our study:

```
FS<-Icex(sur=sur, set=set, planes=list(c(4,5,8),c(3,6,7)), keep=c(TRUE,FALSE), param1=2, param2=NULL, alpha=20, center=NULL, mode=4)
```

It can be applied a reduced landmark configuration (see Figure S2, a in Supporting Information Figures) for the extraction of the orbits only, by changing parameters as follows:

```
FS<-Icex(sur=sur, set="subset", planes=list(c(2,3,6),c(1,4,5)), keep=c(TRUE,FALSE), param1=2, param2=NULL, alpha=20, center=NULL, mode=4)
```

To print the volume of the cavity, type:

```
FS$volume
```

Or simply run:

```
FS
```

Example for the application of *Icv*. It is possible to separately apply *Icv* to a mesh (e.g., a previously extracted and saved COI).

Upload of the mesh:

```
sur<- ply2mesh("name_mesh.ply")
```

Application of *Icv*. The parameter `alpha` must be set according to the desired COI (e.g., in case of PA, `alpha=50`):

```
sur_2<- Icv(sur,alpha=50)
```

The volume of the cavity and the plot of the two meshes are automatically returned. To save the new α-shape, if needed:

```
mesh2ply(sur_2$AlphaShape,"conv_sur")
```

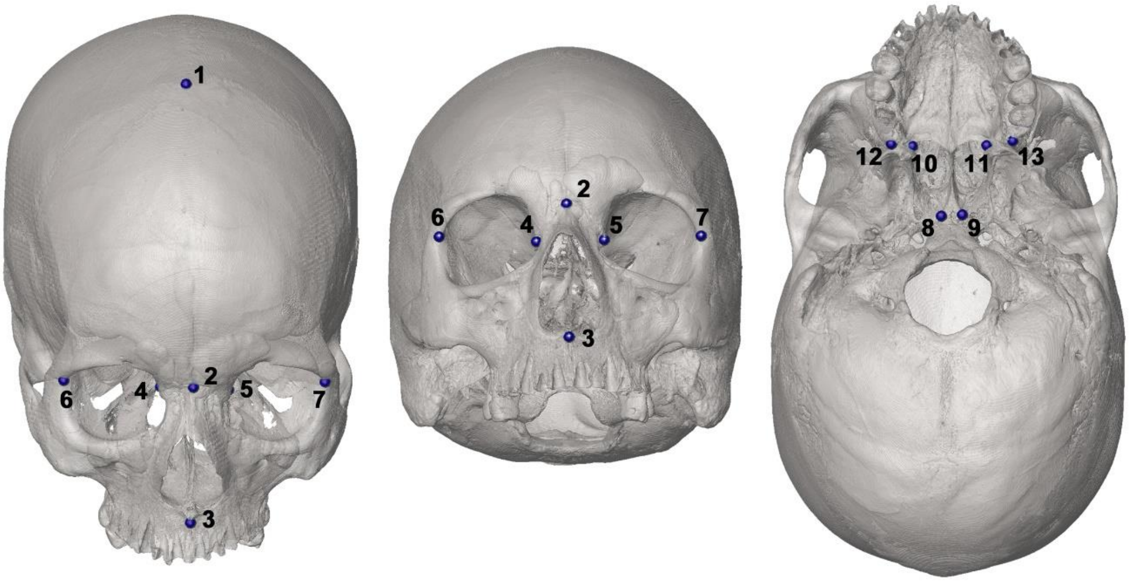

FIGURE S1 Reduced landmark configuration for the extraction of nasal cavity and maxillary sinuses

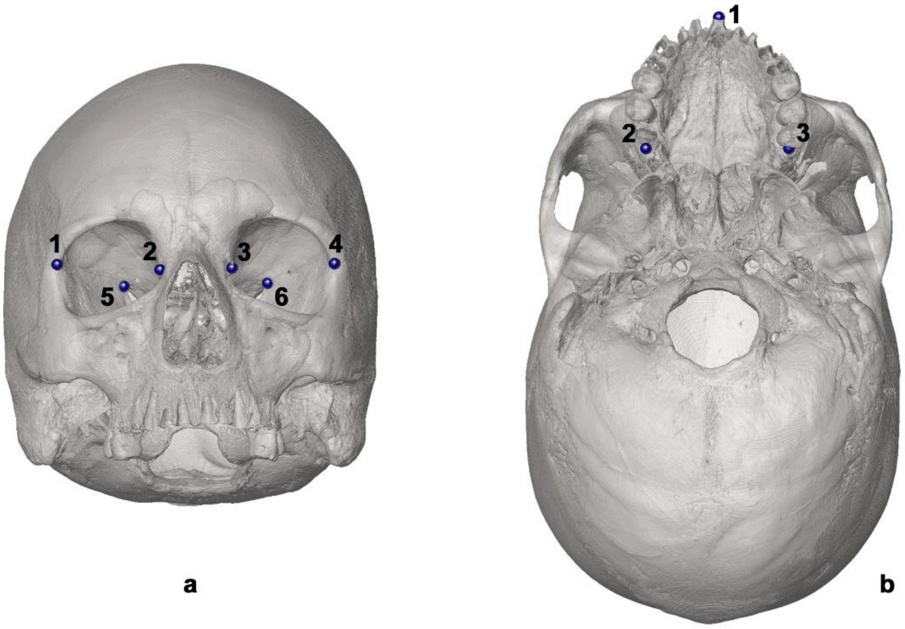

FIGURE S2 Reduced landmark configurations for the extraction of the orbits (**a**) and for the palatal cavity (**b**)

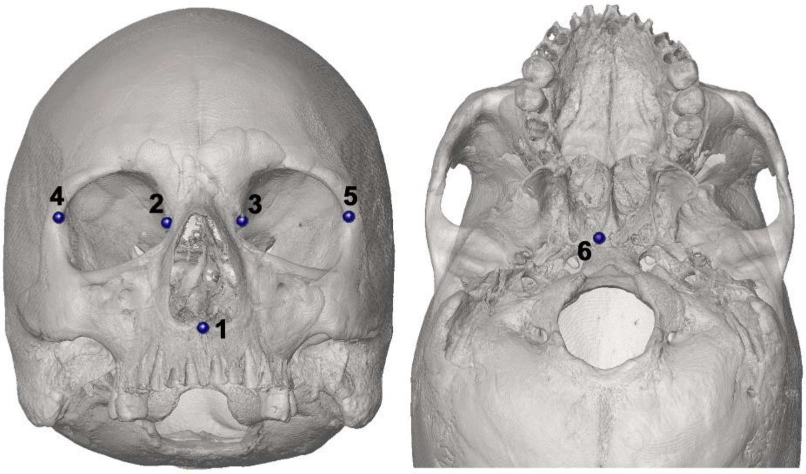

FIGURE S3 Reduced landmark configuration for the extraction of the frontal sinuses

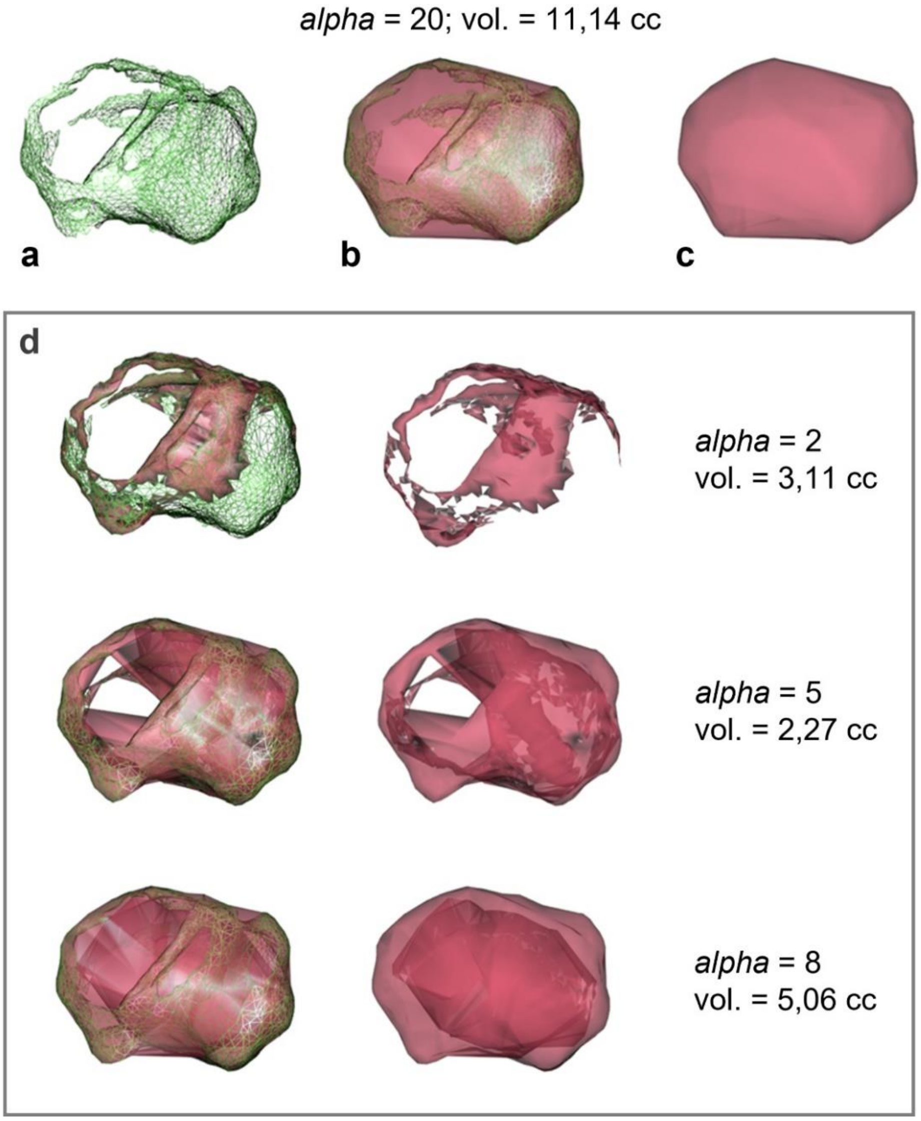

FIGURE S4 Building of the α-shape, by the optimal setting of *alpha*. **a:** extracted cavity (left maxillary sinus, sub-adult); **b:** building of the α-shape; **c:** visualization of the correct α-shape, used for approximating the volume. **d:** incorrect settings of *alpha* and creation of artifacts (e.g., gaps, exclusion of the inner volume from the α-shape) with a subsequent wrong calculation of the volume.

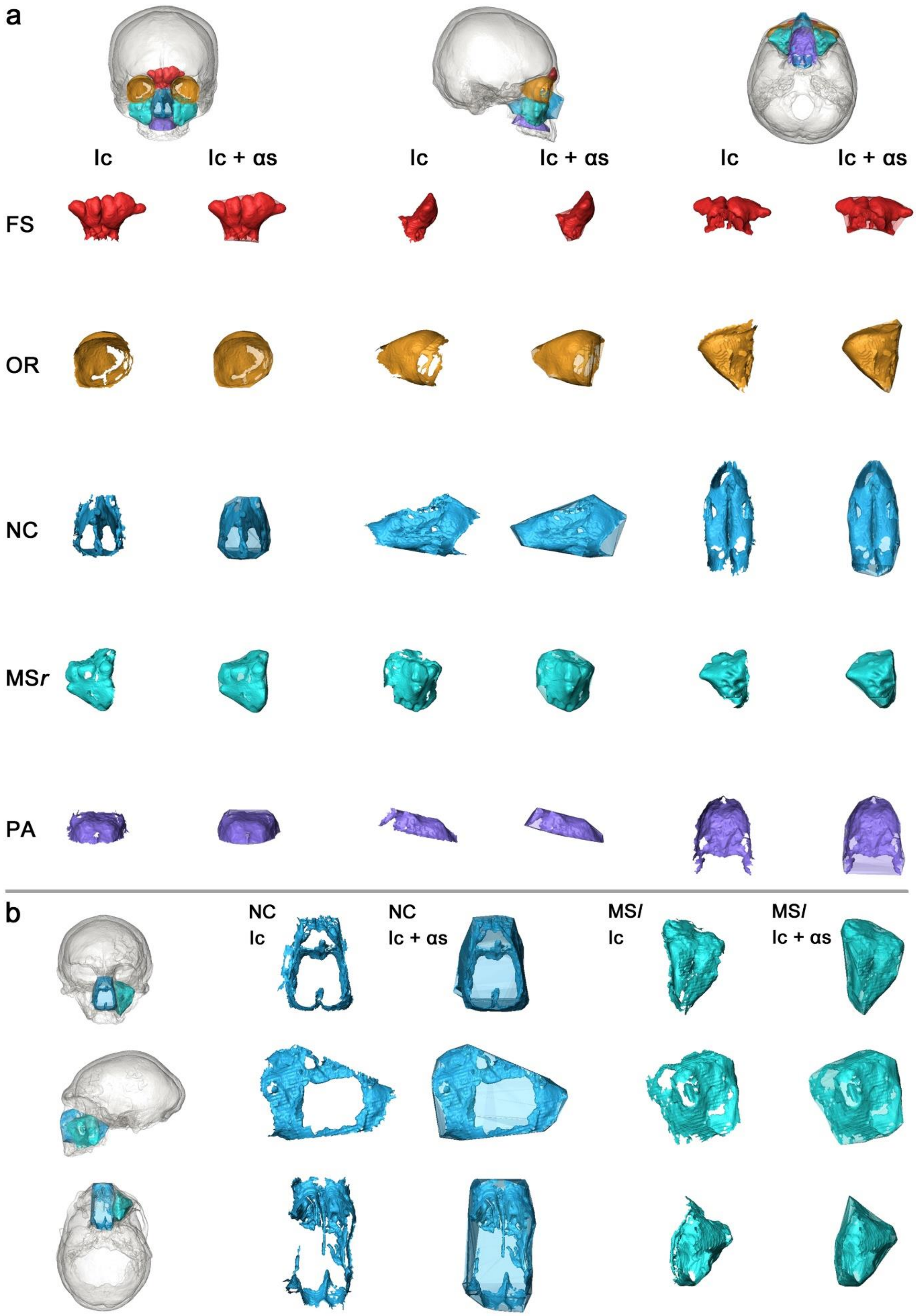

FIGURE S5 Representation of the cavities extracted from a modern individual (male, 19 years, **a**) and Guattari 1 (**b**). **Ic**: inner surface of the cavity; **Ic + αs**: the same inner surface embedded in the α-shape. **FS**: frontal sinuses; **OR**: right orbit; **NC**: nasal cavity; **MS*r***: right maxillary sinus; **PA**: upper oral cavity (or palate); **MS*l***: left maxillary sinus. Cavities and skulls not to scale.

TABLE S1 The sample used for the study. (M): probably male; (F): probably female; Pa_tr_: (*Pan troglodytes*); Go_go_: (*Gorilla gorilla*); Ma_si_: (*Macaca sinica*); NMNH: National Museum of Natural History (Washington, USA); na: the files were obtained as 3D mesh

Individual	Age	Sex	Source	Voxel size (mm)
2DF	2 days	F	Necker - Enfants Malades University Hospital, Paris	0.293 x 0.293 x 0.625
1M11DF	1 month, 11 days	F	Necker - Enfants Malades University Hospital, Paris	0.391 x 0.391 x 0.625
24M21DF	24 months, 21 days	F	Necker - Enfants Malades University Hospital, Paris	0.391 x 0.391 x 0.312
35M24DF	35 months, 24 days	F	Necker - Enfants Malades University Hospital, Paris	0.430 x 0.430 x 0.312
45M7DF	45 months, 7 days	F	Necker - Enfants Malades University Hospital, Paris	0.391 x 0.391 x 0.312
F9_127657	9 years	F	New Mexico Decedent Image Database	0.417969 x 0.417969 x 0.5
M9_195011	9 years	M	New Mexico Decedent Image Database	0.425781 x 0.425781 x 0.5
F14_104011	14 years	F	New Mexico Decedent Image Database	0.447266 x 0.447266 x 0.5
M14_136300	14 years	M	New Mexico Decedent Image Database	0.529297 x 0.529297 x 0.5
F19_115804	19 years	F	New Mexico Decedent Image Database	0.472656 x 0.472656 x 0.5
M19_106561	19 years	M	New Mexico Decedent Image Database	0.488281 x 0.488281 x 0.5
Kabwe	adult	(M)	NESPOS Society Database	0.46875 x 0.46875 x 1
Gibraltar 1	adult	(F)	NESPOS Society Database	0.429687 x 0.429687 x 1
Guattari 1	adult	(M)	NESPOS Society Database	0.486301 x 0.486301 x 1
Pa_tr_220062	adult	F	NMNH - Smithsonian Open Access Database	na
Pa_tr_174704	adult	M	NMNH - Smithsonian Open Access Database	na
Go_go_590951	adult	F	NMNH - Smithsonian Open Access Database	na
Go_go_174712	adult	M	NMNH - Smithsonian Open Access Database	na
Ma_si_271190	adult	F	NMNH - Smithsonian Open Access Database	na
Ma_si_15259	adult	M	NMNH - Smithsonian Open Access Database	na

TABLE S2 List of the arguments of *Icex*

Name	Description
sur	Triangular mesh of the cranium
set	Landmark set
planes	List of matrices specifying the planes
keep	Vector specifying the portion of the mesh to keep at each cut
param1	Numeric parameter for spherical flipping
param2	Numeric parameter for ast3d
alpha	Numeric value of α for the calculation of volume
mode	Indication of the method to be applied (1, 2, 3 or 4)
multiple	Logical value for the keeping of additional portions in case of separated cavities (e.g., FSs)
